# The Relational Impact of Multiple Sclerosis: An Integrative Review of the Literature Using a Cognitive Analytic Framework

**DOI:** 10.1007/s10880-017-9506-y

**Published:** 2017-07-29

**Authors:** Joanna Blundell Jones, Sue Walsh, Claire Isaac

**Affiliations:** 1 Clinical Psychology Unit, Department of Psychology, University of Sheffield, Sheffield, S10 2TN UK; 2 Russell Cairns Unit, Oxford University Hospitals NHS Foundation Trust, Oxford, OX3 9DU UK

**Keywords:** Multiple sclerosis, Relationships, Wellbeing and mental health, Cognitive analytic therapy, Adjustment

## Abstract

This integrative literature review uses cognitive analytic therapy (CAT) theory to examine the impact of a chronic illness, multiple sclerosis (MS), on relationships and mental health. Electronic searches were conducted in six medical and social science databases. Thirty-eight articles met inclusion criteria, and also satisfied quality criteria. Articles revealed that MS-related demands change care needs and alter relationships. Using a CAT framework, the MS literature was analysed, and five key patterns of relating to oneself and to others were identified. A diagrammatic formulation is proposed that interconnects these patterns with wellbeing and suggests potential “exits” to improve mental health, for example, assisting families to minimise overprotection. Application of CAT analysis to the literature clarifies relational processes that may affect mental health among individuals with MS, which hopefully will inform how services assist in reducing unhelpful patterns and improve coping. Further investigation of the identified patterns is needed.

## Introduction

In the United Kingdom, approximately 15 million people live with chronic illness (Department of Health [DOH], 2012). Chronic illness occurs in the context of an individual’s social, domestic and working lives, often causing disruption and increasing mental distress. Social support is considered vital to effective coping, and families have an important role to play in supporting adjustment (Fisher & Weihs, 2000); however, chronic illness can threaten relationships, causing distancing and deterioration (Rolland, 1999), thereby exacerbating levels of distress. Ameliorating relationship breakdown and stress may support positive health outcomes, but to achieve this, coherent understandings of how chronic illness influences relationships are needed. In this paper, the relational consequences occurring for patients with multiple sclerosis (MS) will be explored in more detail, and a cognitive analytic therapy (CAT) framework will then be applied to make explicit the impact of relational changes upon mental health.

MS is a demanding neurological condition, whose symptoms can create a need for social support over a long, and uncertain, trajectory (Gulick, 1994). Symptoms can be many and varied in severity, visibility and presence, and can change over time. MS typically onsets in early adulthood when childrearing and career development are key developmental tasks. As a chronic, unpredictable and progressive condition, MS affects family and social life. Among persons with multiple sclerosis (pwMS), there is a high incidence of comorbid depression and anxiety (Korostil & Feinstein, 2007; Sollom & Kneebone, 2007), and pwMS who believe that MS negatively influences their family life are at higher risk for depression (Leonavičius & Adomaitienė, 2012). Poor negotiation of illness-imposed relational changes may damage the relationships most needed to cope well with MS and subsequently negatively affect the long-term emotional wellbeing of pwMS and their family members. In order to explore these issues further, it is proposed that use of an approach such as CAT, which seeks to understand relational problems and their interaction with wellbeing, may be helpful.

### Cognitive Analytic Therapy

CAT (Ryle, 1995) is a form of psychotherapy concerned with understanding learned patterns of interaction individuals have developed with themselves and with others, and how such patterns connect with psychological distress. This review uses CAT theory and concepts to elucidate relational issues that may affect the medical, social and psychological management of MS. CAT’s focus on mapping out relational sequences enables the development of a clear conceptual organisation of patterns observed. A clear conceptualisation will facilitate transfer of research knowledge into practice to inform care and treatment. CAT is relevant to MS because it has demonstrated efficacy and utility in understanding relational issues and ameliorating distress with a range of health conditions (e.g., asthma: Chapman, Walker, Cluley, & Fabbri, 2000; Walsh, Hagan, & Gamsu, 2000; brain injury: Rice-Varian, 2011; diabetes: Fosbury, Bosley, Ryle, Sonksen, & Judd, 1997; medically unexplained symptoms: Jenaway, 2011) and mental health diagnoses (e.g., anorexia nervosa, anxiety disorders, dementia, depression, personality disorders, psychosis: Ryle & Kerr, 2002). As far as we are aware, this will be the first paper that applies CAT thinking and principles to make clinically relevant sense of published literature on relationships in general, as well as among individuals with MS more specifically.

According to CAT, key relational patterns are learned in early life experiences and form a repertoire that is re-enacted in adult relationships. This repertoire is conceptualised as consisting of *reciprocal roles* (Ryle, 1995). Each reciprocal role is comprised of a parent-derived (powerful) and child-derived (vulnerable) position. Some reciprocal roles are maladaptive, and identifying these brings an opportunity for change and a potential reduction in distress. To be clear, in the context of this review, we are not stating that MS relational patterns are learnt in early childhood; rather, we assert that in the context of living with MS, patterns will often mirror parent–child positions due to the fact that chronic illness often leaves individuals feeling vulnerable and powerless.

Once reciprocal roles are identified, a *sequential diagrammatic reformulation* (SDR; Ryle & Kerr, 2002) is developed. An SDR is a graphic representation of a written formulation that maps out a client’s difficulties and maintaining cycles, i.e., cycles of repeated interaction with the self and others that cause stress and negatively impact wellbeing. SDRs are used to help identify and plan “exits” from unhelpful relational patterns. In a CAT framework, “exit” points are potential opportunities to change behaviour and thinking, which can free a client from being caught up or “trapped” in maladaptive, stress-causing relational roles and behavioural patterns. Through therapy, clients learn to recognize and take advantage of opportunities to exit from and avoid maladaptive cycles of behaviour and thereby enhance functioning and wellbeing.

We will develop an SDR-derived diagram for MS that summarises the literature. Through the use of CAT, we propose that specific unhelpful patterns of relating to others will be revealed that maintain distress, alongside helpful patterns that can have the opposite beneficial effect.

### Aims

This review aims to develop a coherent understanding of how MS influences relational functioning and wellbeing. It applies the conceptual underpinnings of CAT to refine that understanding and to develop a diagrammatic formulation of the patterns identified which will highlight exits from unhelpful patterns. In this way, targets for clinical intervention will be revealed from the literature.

### Integrative Review

In order to develop as rich an understanding as possible of the relationship factors in MS, we used an integrative methodology to review literature on the relationships of individuals with MS, namely how they relate to themselves, their loved ones, and society. Integrative reviews combine evidence from experimental and non-experimental research in order to develop a fuller understanding of a phenomenon of concern (Whittemore & Knafl, 2005). Following the problem identification, literature search and data evaluation stages, the final stages in an integrative review are data analysis and presentation. Data must be extracted, coded and compared in order to identify themes. Data were synthesised using a CAT framework and are presented within that framework.

## Method

### Search Strategy

**Fig. 1:**
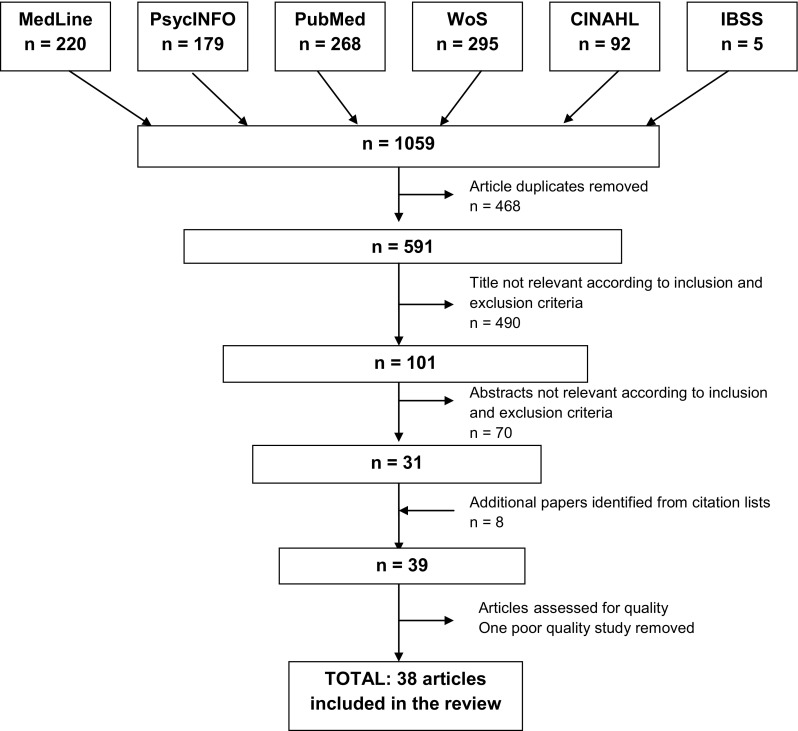
Flow diagram illustrating selection of articles

Database searches were carried out on Ovid MEDLINE(R), Web of Science (WoS), PsycINFO, Cumulative Index to Nursing and Allied Health Literature (CINAHL), PubMed and International Bibliography of the Social Sciences (IBSS) to identify relevant studies on MS. Articles were searched from the inception of each database to July 2014. Relevant articles were also drawn from reference lists or the “Related Citations” function on PubMed. The search strategy and screening process are illustrated in Fig. 1.

The following terms were combined for searching with a limit of “English language only” where possible:

- “Multiple Sclerosis”
- AND (relationships OR impact)
- AND (family OR spouse OR identity OR social support)

Articles were included if they provided specific information about the effect of MS on relationship with sense of self and/or relationships with others and the issues that can arise (thoughts, feelings, behaviours). Excluded publications included those not directly relevant to the topic, medical articles, case studies, book chapters, non-English language publications, and those addressing paediatric MS.

### Data Evaluation

Article quality was assessed using checklists adapted for this particular review from those used by Bogosian, Moss-Morris, and Hadwin (2010). Tables 1 and 2 detail the checklists that were used to assess quantitative and qualitative studies, respectively. For each study, the first author (JBJ) rated each checklist item as either positive or negative; the total number of positives was subsequently calculated, and this score was used to assign an overall rating of good (G), medium (M) or poor (P) quality. Table 3 illustrates the scores required for quality classifications. Twenty-eight studies were classified as good quality and ten as medium. The second and third authors independently rated two randomly selected articles each. These ratings were directly compared with those of the first author. Whilst overall quality ratings did not differ, seven instances of inter-rater disagreement were found across the four articles. These instances were discussed until consensus was achieved. Finally, the original ratings of all remaining articles were rechecked by JBJ, i.e., the presence/absence of criteria was checked.

**Table 1:** Quantitative study quality assessment criteria

Item definition	
Rationale-aims	A: positive if the objective of the study was sufficiently described
Demographic variables	B: positive if information was reported on pwMS gender, age, disease type/course, disease severity, time since diagnosis, current MS status (at least 3 of these) AND if a relative-focused study: their gender, age, nature of their relationship with pwMS as well as the previous
Suitability of the design to answering the research question	C: positive if appropriate research design was used, e.g. positive if control group was used when comparing psychopathology to the healthy population, if cross sectional design was used to find associations among the variables (not suggest causality or predictors), or qualitative methods were used to investigate in depth pwMS’ or relatives’ experiences
	D: positive if control group was equivalent in age, sex and socioeconomic status with the single difference that the person did not have MS (comparative studies only)
	E: positive when analysing different age groups separately when people in a wide age span were studied, or positive when studying a specific age group only
Statistical analysis	F: positive if appropriate statistical methods of analysis were used for the data
Presentation of the analysis	G: positive if the graphs and tables were easy to understand, e.g., presenting a table for regression analyses including *R* ^*2*^ values and β weights
	H: the confidence intervals or *p*-values were given for the main results
Measures used	I: positive if all the questionnaires used were standardized, defined as questionnaires that had been validated and published or psychometric data of new measures were presented
Conclusions	J: positive if the conclusions were justified based on the research findings
Limitations	K: positive if key limitations were mentioned

Based on criteria provided by Bogosian et al. (2010)

**Table 2:** Qualitative study quality assessment criteria

Item definition	
Report explicit scientific context and purpose	A: positive if the manuscript specified where the study fitted within relevant literature and stated the intended purposes or questions of the study
Situating the sample	B: positive if authors described the research participants and their life circumstances to aid the reader in judging the range of people and situations to which the findings might be relevant
Appropriate methods	C: positive if the methods and procedures used were appropriate or responsive to the intended purposes or questions of the study
Specification of methods	D: positive if authors reported all procedures for gathering data, including specific questions posed to participants. Ways of organizing the data and methods of analysis were also specified
Clarity of presentation	E: positive if the manuscript was well-organized and clearly written, with technical terms defined
Grounding in examples	F: positive if authors provided examples of the data to illustrate both the analytic procedures used in the study and the understanding developed in the light of them
Providing credibility checks	G: positive if credibility checks were provided where relevant, these may include (a) checking these understandings with the original informants or others similar to them, (b) using multiple qualitative analysts, (c) comparing two or more varied qualitative perspectives, or (d) where appropriate, “triangulation” with external factors (e.g. outcome or recovery) or quantitative data
Coherence	H: positive if the understanding was represented in a way that achieved coherence and integration while preserving nuances in the data
Appropriate discussion	I: positive if the research data and the understandings derived from them are discussed in terms of their contribution to theory, content, method, and/or practical domains, with limitations acknowledged

Based on criteria provided by Bogosian et al. (2010)

**Table 3:** Quality classifications according to total scores on quality guidelines

Quality classification	Methodological group	
	Quantitative	Qualitative
Good	9–11 points	7–9 points
Medium	6–8 points	4–6 points
Poor	<6 points	<4 points

### Process of Data Extraction, Analysis and Synthesis

Each article was analysed separately for relational processes by JBJ. Relational processes were defined as patterns of relating to self and others as revealed by behaviours, thoughts, and feelings reported in the context of relational interactions. Initially, annotations were made in article margins of words used to describe: the nature of relationships, how individuals were left feeling by others, and how others were experienced as behaving. JBJ subsequently collated the large number of relational words generated. Through discussion, the authors gradually grouped and formed clusters of these relational words, synthesised them, and generated a smaller set of terms that captured major relational themes across all the articles. From this smaller set, pairs of themes were jointly constructed that mirrored CAT reciprocal roles (see Ryle, 1995). Although CAT has a set of common childhood-derived reciprocal role patterns (Ryle & Kerr, 2002), the labels for these reciprocal roles are not fixed, and so they can be adapted to the language of each individual client. JBJ “verified” the final set of pairings by checking it was grounded in and evidenced by article data as each article was read again. Resultant themes and diagrammatic formulation were discussed with a psychologist working therapeutically with pwMS and an accredited CAT practitioner.

### Structure for Data Presentation

In this paper, descriptive and quality data regarding the included articles will be presented first. We will then provide an overview of the article content making reference to the tabular data provided. Finally, we will expound our innovative CAT informed synthesis of the literature, discussing the five common relational responses we have identified, here called reciprocal roles. CAT understands that relational patterns are enacted in intimate and wider relationships, so both will be discussed from the perspectives of pwMS as well as family members. The evidence for the different relational patterns will be presented in turn, and their connection with wellbeing considered with the aid of an SDR-derived diagram.

## Results

### Identified Papers

The literature concerning relationships between pwMS and others (i.e., partners, children, wider family, friends, acquaintances, healthcare professionals, strangers and society as a whole) was considered, and results will be presented using CAT reciprocal roles. Table 4 categorizes the studies in this review according to type of participant studied, e.g., whether pwMS or a specific type of other. Of the six types of participant samples, the majority of studies were pwMS (*n* = 18). Studies also investigated the unique experiences of family members, and some looked at pwMS concurrently with their relatives. Five studies used quantitative methods, obtaining data via surveys and questionnaires; four of these were cross-sectional and one compared questionnaire data at two time points (Pakenham & Cox, 2012; Row 5 of Table 7). The methodology of most of the qualitative studies can be classified as belonging to one of 5 well-known methods (see Table 5 for a brief description of these methods). However, some studies used methods uncommon in psychological research, or did not provide sufficient information in their methods section to allow classification. For example, the method section of Courts, Newton, and McNeal (2005; Row 7 of Table 6) hints at inductive thematic analysis but it is not stated. The most frequently used methodology was inductive thematic analysis (ITA; *n* = 10), followed by interpretative phenomenological analysis and by constant comparative analysis (*n* = 5, respectively). ITA, is a widely-used qualitative analytic method, yet it is notoriously hard to characterise as all qualitative methods are trying to identify themes. In general, ITA involves descriptively “coding” answers for issues of interest to the research question. The approach is “inductive” because the themes that develop from linking codes are intimately bound with the data they represent; labels are not forced onto the data based on theory. As indicated in Table 5, there is substantial overlap with other methodologies. For example, in all methods, to develop broader level themes, initial codes are reviewed and compared with others iteratively.

**Table 4:** Studies that report relational aspects of living with MS

Participants (number of studies in review)	Qualitative	Quantitative
PwMS (18)	Dyck (1995) M Irvine, Davidson, Hoy, and Lowe-Strong (2009) G Galushko et al. (2014) G Koch, Kralik, and Eastwood (2002) G Kosmala-Anderson and Wallace (2013) G Kralik, Koch, and Eastwood (2003) G Malcomson, Lowe-Strong, and Dunwoody (2008) G McClurg, Beattie, Lowe-Strong, and Hagen (2012) G Mozo-Dutton, Simpson, and Boot (2012) G Olsson, Lexell, and Söderberg (2005) M Olsson, Lexell, and Söderberg (2008) G Olsson, Skär, and Söderberg (2011) G Payne and McPherson (2010) G Ploughman et al. (2012) G Reynolds and Prior (2003) G	Green and Todd (2008) G McCabe, McDonald, Deeks, Vowels, and Cobain (1996) G Özdemir and Aşiret (2011) M
Partners (5)	Bogosian, Moss-Morris, Yardley, and Dennison (2009) G Cheung and Hocking (2004) G Courts et al. (2005) G DesRosier, Catanzaro, and Piller (1992) M Mutch (2010) G	
Children of pwMS (4)	Bogosian, Moss-Morris, Bishop, and Hadwin (2011) G Jonzon and Goodwin (2012) G Turpin, Leech, and Hackenberg (2008) M	Pakenham and Cox (2012) G
Relatives (2)	Bowen, MacLehose, and Beaumont (2011) G Hughes, Locock, and Ziebland (2013) G	
Couple experiences (4)	Boeije, Duijnstee, and Grypdonck (2003) G Boland, Levack, Hudson, and Bell (2012) G Esmail, Munro, and Gibson (2007) M Esmail, Huang, Lee, and Maruska (2010) M	
PwMS and relatives (4)	Edmonds, Vivat, Burman, Silber, and Higginson (2007a, b) G Grytten and Måseide (2006) M Power (1985) M	Hakim et al. (2000) M

*PwMS* people with MS, *M* medium quality, *G* good quality

**Table 5:** Commonly used qualitative methodologies

Method (number of studies in review)^a^	Characteristics of method	Additional comment
Inductive thematic analysis (10)	An umbrella term covering methods that aim to identify and describe patterns (themes) across a dataset through a process of data familiarisation, data coding, theme development and theme revision	The research does not have to be connected to a particular theoretical framework or epistemological position—it could be realist or constructivist
Constant comparative analysis (5)	The first interview is coded and then all subsequent interviews are compared to that and to each other. Comparisons continue as codes combine to form larger categories. Data is “fragmented” and then “connected” so that the individual and then the whole is seen	Connected to Grounded Theory (see below) but distinct as no theory is developed
Interpretative phenomenological analysis (IPA) (5)	IPA involves in-depth line-by-line analysis of individual interview transcripts, looking at the language used in order to understand the perspective of the individual. Each interview is analysed separately before links or points of difference are found across cases	Goal of IPA research is to understand the “lived experience” of a particular phenomenon. Developed by Smith, Flowers and Larkin
Hermeneutic phenomenology (4)	Many variations in method exist all with the aim of explaining and understanding the meaning of human experience, primarily through interpretation of narratives. This may consist of three phases: naive reading, structural analysis, comprehensive understanding of the whole text (e.g., Olsson et al., 2005)	Connected with philosophers such as Husserl, Ricoeur and Heidegger
Other (3)	There also exist other lesser known methods of qualitative analysis e.g., Payne and McPherson (2010) use Interpretive Description developed by Thorne. An approach from Nursing, it is characterised by immediate and progressive interview analysis throughout the study e.g., Koch et al. (2002) and Kralik et al. (2003) use the principles of Participatory Action Research developed by Stringer. They use group sessions to discuss the issue of research interest. Preliminary analyses are taken back to the group and discussed	
Grounded theory (2)	A constant comparative method is used to analyse data from interviews in order to develop a theory. Researchers keep interviewing until “data saturation” is reached, i.e. when unique themes no longer appear	Goal of such research is to develop a plausible and useful theory

^a^Four studies included in the review do not clearly state the qualitative method used

**Table 6:** Summary of relevant findings and conclusions from qualitative studies

Number	Author (year)	Aim	Method and sample size	Analysis	Themes reported	Quality rating
1	Boeije et al. (2003)	Explore commitment and caregiving in couples in total care phase	Semi-structured interview 17 couples	Constant comparative analysis	1. Five themes a. The first stages: becoming more experienced in caregiving b. The total care phase c. Inevitability of caregiving d. A shared disadvantage e. Commitment to prevent admission 2. Living with MS involved gradual change, and was a learning process for both partners	7
2	Bogosian et al. (2009)	Explore partner experiences of living with early stages of MS	Semi-structured interview (phone) 15 spouses	Inductive thematic analysis	Seven themes a. Initial reactions to diagnosis b. Loss of control c. Constant worry d. Lifestyle changes e. Social isolation f. Relationship changes g. Attempts to adjust	8
3	Bogosian et al. (2011)	Explore how adolescents adjust to parental MS	Semi-structured interview 15 children	Inductive thematic analysis	Two themes a. Barriers and enhancements to adjustment b. Impact on everyday life	7
4	Boland et al. (2012)	In-depth exploration of couple coping in MS	Semi-structured interview 7 couples	Interpretative phenomenological analysis	Four themes a. Coping together: “Peaks and troughs” b. Coping over the long haul c. Faith in self d. Faith in each other	8
5	Bowen et al. (2011)	Explore experiences of family members following admission of relative with advanced MS	Semi-structured interview 25 relatives	Grounded theory	Four themes a. Information, communication and understanding b. Family relationships, roles and responsibilities c. Emotion, coping and support d. Life outlook and reflection	8
6	Cheung and Hocking (2004)	Explore spousal carers’ experiences of caring for chronically ill partners	Unstructured interview 10 spouses	Hermeneutic phenomenology	One of two major themes (the other is reported in a separate article): Caring as worrying. This has two key subthemes a. Worrying about their partner and their relationship b. Worrying about the future	7
7	Courts et al. (2005)	Investigate lived experience of spouses of pwMS	Focus group 12 spouses	Not stated	Four themes a. Caregiver roles b. Need for resources c. Relational changes d. Barriers	7
8	DesRosier et al. (1992)	Describe experience and coping of wives with housebound husbands	2 focus groups 9 wives	Constant comparative analysis	1. Two themes a. Coping b. The need for Space 2. Women experienced significant personal hardship	6
9	Dyck (1995)	Explore workplace experiences of women with MS	Semi-structured interview 23 women	Not stated	1. Three themes a. Changing lifeworlds b. Changing space c. Restructuring of home and neighbourhood 2. The changing experience of place (issues of access and meaning)	5
10	Edmonds et al. (2007a)	Explore experiences of people severely affected by MS	Semi-structured interview Data relates to 32 pwMS from 23 pwMS and 17 carers	Constant comparative analysis	Three themes a. Physical abilities b. Independence c. Relationships	8
11	Edmonds et al. (2007b)	Explore experiences of people severely affected by MS	Semi-structured interview Data relates to 32 pwMS	Constant comparative analysis	Three themes related to service provision a. Fighting for everything b. Continuity and co-ordination of care c. Information	8
12	Esmail et al. (2007)	Understand impact of female MS on couples’ sexual relationships	Semi-structured interview 6 couples	Inductive thematic analysis	1. Six themes from women with MS a. Communication b. Patterns of denial and acceptance c. Impact on sex d. Partner’s needs e. Role changes f. Love and support 2. Five themes from male partners a. Communication b. Impact on sex c. Role change d. Intimacy and closeness e. Partner’s emotional response to MS	6
13	Esmail et al. (2010)	Understand impact of male MS on couples’ sexual relationships	Semi-structured interview 4 couples	Inductive thematic analysis	Four themes a. Communication is important b. MS affected sexual relationship c. Role changes d. Acceptance of MS supports the relationship	6
14	Galushko et al. (2014)	Explore unmet needs in those severely affected by MS	Semi-structured interview 15 pwMS	Constant comparison analysis	Four themes a. Support of family and friends b. Health care services c. Managing everyday life d. Maintaining biographical continuity	8
15	Grytten and Måseide (2006)	Explore stigma experienced by pwMS in social relationships	Semi-structured interview 8 pwMS 6 relatives	Grounded theory	1. Two social processes were identified a. Ignoring illness b. Overemphasising illness 2. These processes impacted social networks and coping	6
16	Hughes et al. (2013)	Explore how people identify with a “carer” role	Narrative interviews 27 partners 2 siblings 5 children 4 parents 2 friends	Inductive thematic analysis	1. Six categories of caring tasks were identified a. Emotional support b. Personal care c. Physical care d. Household tasks e. Advocacy f. Activism 2. Becoming a “carer” was influenced by increasing care needs. The label of “carer” could be embraced, enforced, absorbed or rejected by family and friends	8
17	Irvine et al. (2009)	Explore living with and adjusting to MS	Focus group 8 pwMS	Interpretative phenomenological analysis	Six themes a. Reaction to/impact of being diagnosed b. Social activity c. Role in society and self-worth d. Relationships and dependency e. Attitudes/reactions of others f. Perceptions of adjustment and changes in self-concept, identity and outlook	7
18	Jonzon and Goodwin (2012)	Understand play experiences of daughters who were caregivers to mothers with MS	Semi-structured interview 4 daughters 3 in focus group	Interpretative phenomenological analysis	1. Three themes a. Being a good daughter b. Blurred relationship boundaries c. Encumbered play 2. Daughters’ leisure time was impacted by their mother’s MS. They worried about their mothers and becoming a caregiver meant their own needs could be neglected	8
19	Koch et al. (2002)	Explore how women experience and construct sexuality	5 focus group sessions 12 pwMS 9 semi-structured interviews	Other	Three themes a. Appearance (looking and feeling good themselves) b. Acknowledgement (feeling valued and acknowledged by others) c. Communication (with partners regarding sexual changes)	7
20	Kosmala-Anderson and Wallace (2013)	Explore childbearing experiences of UK women with MS	Semi-structured interview 9 pwMS	Inductive thematic analysis	Three themes a. Concerns about MS and pregnancy b. Lack of information about MS and pregnancy c. Others’ opinions about childbirth choices	8
21	Kralik et al. (2003)	Further exploration of transition in chronic illness and the relationship between self and body	5 focus group sessions 12 pwMS 9 semi-structured interview	Other	1. Two themes a. Extraordinariness b. Ordinariness 2. Exemplars Julie and Lisa illustrate how women’s lives are shaped by illness-related identity shifts. A changed body affects the sense of self, roles, and relationships	8
22	Malcomson et al. (2008)	Explore experiences of people who feel able to cope with MS	2 focus groups 13 pwMS	Inductive thematic analysis	Seven themes a. Something is wrong b. Getting a name c. Getting help d. Consequences in lifestyle e. Getting on with day-to-day life f. Advice to others with MS g. Advice to professionals	7
23	McClurg et al. (2012)	Examine effect of constipation on the quality of life of pwMS	Semi-structured interview 12 pwMS	Inductive thematic analysis	1. Themes a. Loss of normal identity b. Daily impact c. Reluctance of patients and healthcare professionals to discuss bowel problems d. Loss of control 2. A spoiled normal identity and decreased self-esteem due to reactions of others and associated aesthetic issues of bowel dysfunction was found	9
24	Mozo-Dutton et al. (2012)	In-depth exploration of personal perceptions of self and perceived impact of MS on self	Semi-structured interviews 12 pwMS	Interpretative phenomenological analysis	Three themes a. ‘My body didn’t belong to me’: The changing relationship to the body b. ‘I miss the way I feel about myself’: The changing relationship to self c. ‘Let’s just try and live with it’: Incorporating MS within self	8
25	Mutch (2010)	Understand experiences of partners caring for disabled spouse, and explore coping strategies	Semi-structured interview 8 partners	Not stated	1. Five themes a. Worry b. Planning c. Frustration d. Commitment to marriage e. Coping strategies	8
26	Olsson et al. (2005)	Explore what fatigue means to women with MS	Semi-structured interview 10 pwMS	Hermeneutic phenomenology	1. Two themes a. Experiencing the body as a barrier b. Experiencing a different absence 2. Subthemes cover issues such as being unable to participate and saving strength	6
27	Olsson et al. (2008)	Describe meaning of women’s experiences of living with MS	Semi-structured interview 10 pwMS	Hermeneutic phenomenology	1. Two themes a. An unrecognizable body b. Trying to maintain power 2. Subthemes cover issues such as dependence, feeling seen differently, feeling ignored and wanting to fight as long as possible	8
28	Olsson et al. (2011)	Explore meanings of being received and met by others by women with MS	Semi-structured interview 15 pwMS	Hermeneutic phenomenology	1. Two themes a. Experiencing oneself as a valuable person b. Experiencing oneself as diminished 2. Women sometimes felt needed and appreciated. They also felt they were seen differently, pitied, misunderstood and a burden	8
29	Payne and McPherson (2010)	Explore the experience of motherhood in MS	Semi-structured interview 9 pwMS	Other	Six themes a. Public private experience b. Keeping baby safe c. Enlisting support d. Conserving energy e. Being the ideal mother f. Backgrounding MS	7
30	Ploughman et al. (2012)	Describe experience of ageing with MS	Semi-structured interview 18 pwMS	Inductive thematic analysis	Three themes a. MS recognition process b. The MS experience c. Moving toward self-management	9
31	Power (1985)	Identify key family variables influencing adjustment of pwMS	Semi-structured interview 49 families 80% of families seen twice	Not stated	1. Families were classified as positively adjusted (n = 23) or maladjusted (n = 26) 2. Many factors contributed to positive adjustment including sharing responsibilities and accepting any help offered 3. In the ‘maladjusted’ families, MS was seen as “an everpresent source of trouble,” and good communication and understanding were lacking	4
32	Reynolds and Prior (2003)	Explore women’s strategies for negotiating quality of life in MS	Semi-structured interview 27 pwMS	Interpretative phenomenological analysis	Six themes a. Managing illness and limiting its impact b. Maintaining and extending meaningful roles c. Maintaining mutual relationships d. Clarifying personal beliefs and aspirations e. Dealing with social barriers f. Consciously valuing and promoting the positive	8
33	Turpin et al. (2008)	Explore experience and coping strategies of children with an MS parent	Semi-structured interview 8 children	Inductive thematic analysis	Three themes a. Changing roles and responsibilities b. Emotional impact c. Things that helped	7

*PwMS* people with MS

**Table 7:** Summary of relevant findings and conclusions from quantitative studies

Number	Author (year)	Aim	Design and sample size	Analysis	Key findings	Quality rating
1	Green and Todd (2008)	Examine social and economic impact of MS	Questionnaire 920 pwMS	Descriptive statistics Pearson Chi square Thematic analysis	1. Three-quarters of the respondents felt an impact in at least some of the 8 questionnaire domains. In particular, 49.3% reported an impact on their children, 55.2% on intimate relationships, and 80% on social life and their own employment 2. The impact of MS on all domains increases as disability progresses 3. Two qualitative themes emerged from the open-ended questions: restricting choices, and limiting independence	9
2	Hakim et al. (2000)	Assess social impact of MS and patients’ abilities to fulfill roles	Survey 305 pwMS 223 relatives	Descriptive statistics Mann–Whitney U	1. MS disease severity was associated with employment status and levels of social activity 2. 37% of the sample of pwMS reported a decline in living standards 3. 36% of carers’ reported their careers were affected	6
3	McCabe et al. (1996)	Assess perceived impact of MS on sexual functioning, social and intimate relationships	Questionnaire 111 pwMS	Descriptive statistics ANOVAs Pearson’s *r*	1. Two-thirds indicated that sexual interactions were less frequent 2. Illness duration and level of disability were not predictive of relationship quality, although some participants did report relational changes	8
4	Özdemir and Aşiret (2011)	Identification of economic, family, social, and employment issues of pwMS in Turkey	Questionnaire 101 pwMS	Descriptive statistics Chi square test and independent samples *t*-test	1. 71.3% of the sample reported decreased social activity 2. 49.5% experienced household problems (e.g. communication issues, overprotective family) 3. More MS symptoms were associated with greater difficulties at home, in employment and socially	7
5	Pakenham and Cox (2012)	Explore caregiving in children of a parent with MS	Questionnaires 88 families (85 parents with MS 55 partners 130 children)	Descriptive statistics Factor analysis of Youth Activities of Caregiving Scale (YACS) Hierarchical regression analyses	1. Higher levels of instrumental and social-emotional care tasks were associated with poor adjustment, whereas higher levels of personal-intimate were associated with better adjustment. Domestic-household tasks were unrelated to adjustment 2. Increased levels of caregiving were associated with decreased life satisfaction, increased somatisation and increased total difficulties for children of pwMS	9

*PwMS* people with MS

There were no clear differences between the data provided by good versus medium quality studies. Additionally, there were no clear differences between the data provided by quantitative versus qualitative studies, although the qualitative studies do provide more detailed information on the lived experiences of pwMS and their loved ones. However, we will not focus further on any differences between quantitative and qualitative studies; the purpose of this integrative study is to combine and synthesize information from studies of both types rather than focus on differences between them.

### Key Findings

The findings displayed in Tables 6 and 7 highlight that MS can negatively affect independence by creating a need for care over a long period of time. Although the nature of the extra care needed may vary, there was clear evidence that roles changed; MS meant partners and children became “caregivers” (In Table 6 see: Row 13, Esmail et al., 2010; Row 16; Hughes et al., 2013; Row 17; Irvine et al., 2009; Row 18; Jonzon & Goodwin, 2012; Row 19; Koch et al., 2002; see also Row 1 of Table 7; Green & Todd, 2008). In romantic relationships, partners no longer offered and received care equally, and mutuality, that is a sense of shared activities, values, and emotional closeness (Park & Schumacher, 2014), could be lost. Not only was this change challenging for loved ones, but pwMS felt they were a burden (In Table 6 see: Row 12, Esmail et al., 2007; Row 26; Olsson et al., 2005). Many domains of life changed, or were affected, and such effects were particularly influenced by symptom severity. The literature revealed families and pwMS could respond in different ways to this increasing need for care, and these data are drawn upon for the conceptual CAT analysis.

### Using CAT to Understand Reciprocal Roles

**Fig. 2:**
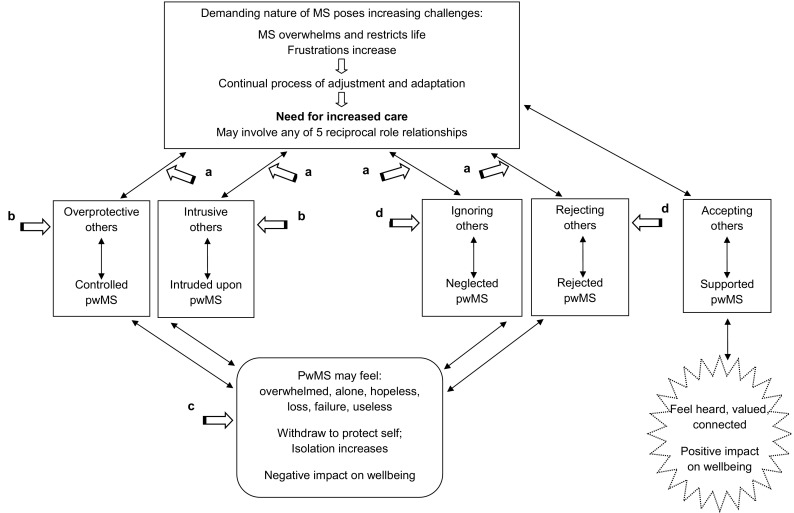
Simplified sequential diagrammatic reformulation (SDR) summarising key relational themes in MS

Five common reciprocal roles were identified: *over protective–controlled; intrusive–intruded upon; ignoring–neglected; rejecting–rejected* and *accepting–supported*. Each role comprises a powerful “doing” and a vulnerable “done to” position. Rather than presenting a diagram typical within therapeutic sessions, Fig. 2 provides a simplified CAT-informed sequential diagrammatic reformulation more suitable for readers unfamiliar with CAT. A more traditional SDR is available on request from the first author. The SDR-derived diagram in Fig. 2 summarises key relational themes in MS and demonstrates how reciprocal roles may be linked with pwMS’ mood and wellbeing. Whilst the focus in the diagram is on consequences for pwMS, in the following text we note experiences of significant others too.

The uppermost rectangle in Fig. 2 summarizes challenging features that MS poses for pwMS and their family members and caregivers. The middle cross-section of Fig. 2 displays five rectangles representing the five reciprocal role relationships identified between pwMS and others (that are also enacted with oneself) that are important for understanding the psychosocial consequences of MS. The lower third of Fig. 2 displays pwMS’ emotional and behavioural responses to four reciprocal role patterns leading to potentially negative psychological consequences for pwMS, and one relational pattern with more beneficial consequences. The thin-line arrows that form connective paths between components of Fig. 2 are shown as two-sided arrows to highlight the bidirectional causal paths that are considered likely to characterize the relationships between components shown in Fig. 2. The large arrows with superscripts depict possible exit points from unhelpful interaction cycles, which also are potential points for professional intervention. This component of Fig. 2 will be further explained at the end of the "Results" section.

#### Overprotective–Controlled Reciprocal Role

Dependency was uncomfortable for many pwMS, even infuriating (In Table 6 see: Row 9, Dyck, 1995; Row 10; Edmonds et al., 2007a; Row 17; Irvine et al., 2009; Row 32; Reynolds & Prior, 2003; In Table 7 see Row 1; Green & Todd, 2008). Although partners were considered vital for support (physical, emotional, financial), MS-induced changes in dependence meant care could become overbearing, which caused relational strain and tension. The influence and/or presence of MS could become overemphasised by family members (Grytten & Måseide, 2006; Row 15 of Table 6), and when this happened, pwMS felt more ill (see also Olsson et al., 2011; Row 28 of Table 6). They felt infantilised and pitied, that the expectations others had of them were low and that they were no longer given responsibility (In Table 6 see: Row 27, Olsson et al., 2011; Row 31; Power, 1985). Partners of pwMS desired to be supportive, but partners also recognised they could be hypervigilant to difficulties and overprotective (Courts et al., 2005; Row 7 of Table 6). PwMS believed partners did not like watching them struggle with tasks, and so would intervene prematurely. Overinvolvement of family could lead pwMS to withdraw, which resulted in isolation (Grytten & Måseide, 2006; Row 15 of Table 6) and added to overwhelming feelings. Overprotection seemed to be either a family’s attempt to be supportive, or a method for managing their own anxiety. Rather than being experienced as supportive by pwMS, well-meaning interventions often had an opposite effect; when families were overprotective, pwMS felt controlled or minimised.

#### Intrusive–Intruded Upon Reciprocal Role

MS intruded into the lives of pwMS, their friends and family. A number of aspects of MS were experienced as intrusive, demanding and overwhelming, not least the unpredictability of symptoms. Women expressed feeling that MS “had captured” their body, which had become untrustworthy and left them feeling powerless (Olsson et al., 2008; Row 27 of Table 6). Concerns about managing particularly troublesome symptoms like fatigue and bowel dysfunction invaded everyday life; social events or excursions had to be planned (In Table 6 see: Row 22, Malcomson et al., 2008; Row 23; McClurg et al., 2012), and life could no longer be spontaneous (Mozo-Dutton et al., 2012; Row 24 of Table 6). PwMS could not participate as before; employment was restricted and roles that helped form identity could not be performed (In Table 6 see: Row 9, Dyck, 1995; Row 14; Galushko et al., 2014; Row 24; Mozo-Dutton et al., 2012; in Table 7 see Row 2; Hakim et al., 2000), which was accompanied by a sense of loss. Assistance was necessary at times to manage symptoms, but this intrusion into personal space by services could be difficult (In Table 6 see: Row 5, Bowen et al., 2011; Row 9; Dyck, 1995; Row 27; Olsson et al., 2008).

MS also affected motherhood, even intruding into decisions about becoming a parent (Table 6 see: Row 20, Kosmala-Anderson & Wallace, 2013; Row 29; Payne & McPherson, 2010). Women reported a tension between wanting to be an “ideal mother” and needing to conserve energy to look after their own health (Payne & McPherson, 2010; Row 29 of Table 6). Many parents were acutely aware that their children’s educational performance and wellbeing were affected by parental MS (Green & Todd, 2008; Row 1 of Table 7). When MS intruded on their ability to be a “good mother” or fulfill their duties, women were left feeling guilty and devastated (In Table 6 see: Row 26, Olsson et al., 2005; Row 32; Reynolds & Prior, 2003). Women did not want to lose their care provider role (Payne & McPherson, 2010; Row 29 of Table 6), but MS posed “an ever present threat of turning partners and children into caregivers” (Reynolds & Prior, 2003, p. 1236; Row 32 of Table 6).

Symptoms meant pwMS required greater levels of assistance, yet requests for support could be perceived as demanding by family members. Increased responsibilities, especially social-emotional and instrumental tasks, could overwhelm children, and the children’s needs could be overlooked as they had to assume parental or adult-like roles. Caregiving encroached upon play, and guilt and worry made it hard for children to enjoy life at times (In Table 6 see: Row 18, Jonzon & Goodwin, 2012; Row 33; Turpin et al., 2008; In Table 7 see Row 5; Pakenham & Cox, 2012). MS was also a constant source of worry for partners and other relatives (In Table 6 see: Row 2, Bogosian et al., 2009; Row 6; Bowen et al., 2011; Row 6; Cheung & Hocking, 2004; Row 25; Mutch, 2010). Relatives shared their own feelings and problems less, and the relatives’ own needs became side-lined (In Table 6 see: Row 1, Boeije et al., 2003; Row 2; Bogosian et al., 2009; Row 25; Mutch, 2010). Partners felt they lost control over their lives; they needed space yet often suffered in silence as social support felt “out of reach.” These feelings were overwhelming for family members (In Table 6 see: Row 1, Boeije et al., 2003; Row 7; Courts et al., 2005; Row 8; DesRosier et al., 1992; Row 13; Esmail et al., 2010). MS dictated partners’ social lives as activities were planned to accommodate physical symptoms, or did not happen (In Table 6 see: Row 2, Bogosian et al., 2009; Row 4; Boland et al., 2012; Row 7; Courts et al., 2005).

#### Ignoring–Neglected Reciprocal Role

When symptoms limited participation and care needs increased, pwMS reported that friendships “drifted,” and activities that were once shared were no longer enjoyed together, resulting in them feeling “left behind,” neglected and separated from others (In Table 6 see: Row 14, Galushko et al., 2014; Row 19; Koch et al., 2002; Row 24; Mozo-Dutton et al., 2012; Row 27; Olsson et al., 2008; Row 30; Ploughman et al., 2012). Social interactions could leave pwMS feeling unimportant, under scrutiny and disbelieved (Olsson et al., 2011; Row 28 of Table 5); pwMS felt others lacked awareness about the impact of “hidden” yet debilitating symptoms like fatigue (Green & Todd, 2008, Row 1 of Table 7; Olsson et al., 2005, Row 27 of Table 6). Sometimes families denied or ignored the existence of MS and/or its consequences, even refusing to talk about the illness (In Table 6 see: Row 28, Olsson et al., 2011; Row 31; Power, 1985; In Table 7 see: Row 4; Özdemir & Aşiret, 2011). This “violation of self” (Grytten & Måseide, 2006, p. 200; Row 15 of Table 6) left pwMS feeling invalidated and negatively impacted pwMS’ wellbeing. The reason for others’ refusal to acknowledge MS was not stated, but it may relate to the intrusive nature of MS; perhaps they wish to avoid being burdened by complaints and requests for help, or they may desire to minimise embarrassment by not drawing attention to points of difference such as visible MS symptoms or obvious changes in abilities.

Loved ones of pwMS also felt that friends and family did not recognise or understand what they face (In Table 6 see: Row 1, Boeije et al., 2003; Row 2; Bogosian et al., 2009; Row 5; Bowen et al., 2011; Row 7; Courts et al., 2005; Row 18; Jonzon & Goodwin, 2012). Children of parents with MS reported others have minimised their experience of having to cope with a parent’s MS (Bogosian et al., 2011; Row 3 of Table 6), and caregiver daughters noted their own needs felt invisible; they wanted more support and acknowledgement of their role (Jonzon & Goodwin, 2012; Row 18 of Table 6).

PwMS and caregivers felt ignored and neglected by services; they felt they had to “fight for everything” in relation to accessing care; waits were too long, concerns were not taken seriously and consultations were too short (In Table 6 see: Row 11, Edmonds et al., 2007b; Row 14; Galushko et al., 2014). Staff changes, service inconsistency and inflexibility, alongside a lack of coordinated care, exacerbated the feeling that their needs were neglected (In Table 6 see: Row 6, Cheung & Hocking, 2004; Row 11; Edmonds et al., 2007b; Row 30; Ploughman et al., 2012). At times, health and social care staff lacked empathy and were “useless” or poorly trained in dealing with MS (In Table 6 see: Row 2, Bogosian et al., 2009; Row 6; Cheung & Hocking, 2004), leaving relatives worried, frustrated, and reluctant to request support (In Table 6 see: Row 6, Cheung & Hocking, 2004; Row 30; Ploughman et al., 2012). Relevant reliable information about MS, especially related to specific concerns like childbearing and bowel dysfunction, was desperately wanted, but pwMS and caregivers felt it was not available and that they were deserted, unsupported and “fobbed off” by health professionals (In Table 6 see: Row 5, Bowen et al., 2011; Row 11; Edmonds et al., 2007b; Row 14; Galushko et al., 2014; Row 20; Kosmala-Anderson & Wallace, 2013; Row 22; Malcomson et al., 2008; Row 23; McClurg et al., 2012; Row 26; Olsson et al., 2008, 2011; Row 27; Row 30; Ploughman et al., 2012). PwMS expressed reluctance to raise the issue of bowel dysfunction, feeling that it was a “dirty secret” even in a medical setting (McClurg et al., 2012, p. 16; Row 23 of Table 6). Having experiences being ignored and invalidated left pwMS and their families feeling neglected, powerless and even hopeless.

#### Rejecting–Rejected Reciprocal Role

Many changes brought by MS were resented (In Table 6 see: Row 16, Hughes et al., 2013; Row 31; Power, 1985; Row 33; Turpin et al., 2008); in particular, family members wanted to resist the caregiver role enforced by MS, and to assert and retain their identity as a husband, wife, or daughter (In Table 6 see: Row 1, Boeije et al., 2003; Row 16; Hughes et al., 2013; Row 18; Jonzon & Goodwin, 2012). Familial responses to changed abilities, the changed need for care, and the intrusive and overwhelming nature of MS could be experienced by pwMS as hostile or unkind (In Table 6 see: Row 21, Kralik et al., 2003; Row 31; Power, 1985). Such circumstances can exacerbate feelings of conflict and distancing in a relationship, especially when partners have different coping styles (Boland et al., 2012; Row 4, Table 6). While outright “rejection” by family was rarely reported, MS has been associated with relationship breakdown (In Table 6 see: Row 13, Esmail et al., 2010; Row 14; Galushko et al., 2014; In Table 7 see Row 3; McCabe et al., 1996). MS affects sexual functioning (see Schmidt, Hofmann, Niederwieser, Kapfhammer, & Bonelli, 2005 for a review), and women reported that partners do not understand their experience of sex in the context of MS (e.g., Esmail et al., 2007; Row 12 of Table 6), which caused a relational barrier.

The literature revealed that pwMS could reject themselves. MS-related bodily changes, especially those that are visible, can “violate” one’s sense of personal dignity (Olsson et al., 2008; Row 27 of Table 6). Such changes could seriously shake self-esteem, and self-confidence, and caused some pwMS to feel they are not the same person they once were (In Table 6 see: Row 17, Irvine et al., 2009; Row 21; Kralik et al., 2003; Row 22; Malcomson et al., 2008; Row 23; McClurg et al., 2012; Row 27; Olsson et al., 2008; In Table 7 see Row 1; Green & Todd, 2008). A body-self separation has been reported with pwMS seeing their body as an adversary that could no longer be relied upon; individuals felt useless and like a “failure” (In Table 6 see: Row 24, Mozo-Dutton et al., 2012; Row 27; Olsson et al., 2008). Furthermore, pwMS who were interviewed about bowel dysfunction (McClurg et al., 2012; Row 23 of Table 6) viewed their body as having let them down and stigmatised them; their body became a source of disgust, which influenced their readiness to engage in social interaction. They feared derision and embarrassment if they had a bowel accident, and therefore avoided going out (McClurg et al., 2012).

PwMS’ social involvement was influenced by the attitudes of others, and not just the logistics of organising trips; pwMS felt others seemed uncomfortable or embarrassed in their presence (Green & Todd, 2008, Row 1 of Table 7; Irvine et al., 2009, Row 17 of Table 6). Women felt they would be and were avoided or ignored (Olsson et al., 2008, Row 27 of Table 6), and family noticed a reluctance or refusal to socialise (In Table 6 see: Row 2, Bogosian et al., 2009; Row 31; Power, 1985). Half of Özdemir and Aşiret’s (2011; Row 4 of Table 7) participants felt uncomfortable socially, feeling anxious, insecure, jealous, ashamed and worthless. While pwMS may reject interactions with others to protect themselves, there were also physical barriers which precluded involvement in social activities, such as poor wheelchair access (In Table 6 see: Row 7, Courts et al., 2005; Row 14, Galushko et al., 2014; Row 32; Reynolds & Prior, 2003; In Table 7 see: Row 4; Özdemir & Aşiret, 2011). Reynolds and Prior (2003; Row 32 of Table 6) identified social discrimination and stigmatization as common features of living with MS; pwMS expressed anxiety about using devices such as wheelchairs as others can relate to the disability instead of the person (Ploughman et al., 2012; Row 30 of Table 6). Adolescent children reported frustration with how others treated their MS parent, e.g., staring, patronising, completely ignoring (Bogosian et al., 2011; Row 3 of Table 6). PwMS expressed feeling rejected by a society that values individual contributions; they felt they had lost “normal” adult status and did not have the same worth as others (In Table 6 see: Row 27, Olsson et al., 2008, 2011; Row 28; Row 32; Reynolds & Prior, 2003). While families may channel their frustration into advocacy or activism (Hughes et al., 2013; Row 16 of Table 6), pwMS can be left feeling dejected and wanting to disengage.

#### Accepting–Supported Reciprocal Role

The unhelpful patterns of relating noted above emerge from the increased need for care, but dysfunction is not the whole story; pwMS also report positive relational outcomes. The literature revealed one key helpful reciprocal role pattern, accepting–supported, and as shown in the right-hand side of Fig. 2, this reciprocal role can positively affect wellbeing.

The caregiver role was embraced by some loved ones (Hughes et al., 2013; Row 16 of Table 6), and gender differences were observed in how this manifested itself. Men were “protectors” and “enablers” helping their wives conserve energy, making sure their wives engaged in activities that promoted self-worth, such as helping them be mothers and manage parental responsibilities (In Table 6 see: Row 7, Courts et al., 2005; Row 29; Payne & McPherson, 2010). Women were “advocates,” obtaining necessary supports while encouraging their husband’s independence, and keeping their husbands involved (see also Bogosian et al., 2009; Row 2 of Table 6). Both sorts of behaviour appeared accepting and encouraging, but the perceptions of pwMS were not investigated. In the face of MS-related adversity, couples found they could still admire and respect each other, work as a team, feel committed to each other, that they were in it together, and had become better communicators (In Table 6 see: Row 4, Boland et al., 2012; Row 13; Esmail et al., 2010; Row 25; Mutch, 2010). PwMS felt useful and involved through contributing and trying to help whenever possible, assisted by loved ones being open to re-negotiating task allocation (In Table 6 see: Row 1, Boeije et al., 2003; Row 4; Boland et al., 2012; Row 16; Hughes et al., 2013; Row 29; Payne & McPherson, 2010; Row 31; Power, 1985). Supporting each other brought balance back into relationships and facilitated ongoing participation in family life, which was hugely valued by pwMS.

Positive reactions and affirmation from loved ones were a highly valued source of hope that enabled pwMS to cope with MS (In Table 6 see: Row 17, Irvine et al., 2009; Row 21; Kralik et al., 2003; Row 22; Malcomson et al., 2008). Understanding and acceptance in the face of changes in sexual functioning also was very important (In Table 6 see: Row 12, Esmail et al., 2007, 2010; Row 13). Women felt changed in the eyes of their partners, e.g., “I’m not the girl he married,” and they needed to feel valued regardless of MS (In Table 6 see: Row 17, Irvine et al., 2009; Row 19; Koch et al., 2002). Self-acceptance was challenging for pwMS, but engaging in activities that provided a sense of personal continuity was helpful, and slowly, the self was re-negotiated with MS integrated as one part of the self (In Table 6 see: Row 24, Mozo-Dutton et al., 2012; Row 32; Reynolds & Prior, 2003). Over time the changed body was accepted, and recognising its frailty, pwMS nurtured and worked with their body and dealt with limitations imposed by MS (Kralik et al., 2003; Row 21 of Table 6). PwMS managed their symptoms, and were able to do important things like being with family; mothers found creative solutions to cope with any MS-imposed limitations (Payne & McPherson, 2010; Row 29 of Table 6). Acceptance was not synonymous with “giving in” or “giving up.” Instead, acceptance meant constantly adjusting and adapting while trying to keep life as normal as possible; it meant living with MS while maintaining a sense of fighting it (In Table 6 see: Row 12, Esmail et al., 2007; Row 14; Galushko et al., 2014; Row 24; Mozo-Dutton et al., 2012; Row 27; Olsson et al., 2008; Row 29; Payne & McPherson, 2010; Row 30; Ploughman et al., 2012; Row 32; Reynolds & Prior, 2003).

Accepting MS also meant asking for and letting others help, which was a proactive choice “to take part in life” (Olsson et al., 2008, p. 423; Row 27 of Table 6). Support from services helped maintain independence, and access to an array of providers gave security (In Table 6: Row 9, Dyck, 1995; Row 30; Ploughman et al., 2012). Two-way communication with health professionals was vital, and while some literature suggests that such positive experiences may be in a minority (Malcomson et al., 2008; Row 22 of Table 6), two-way communication with professionals left pwMS feeling reassured, listened to, and taken seriously (In Table 6: Row 20, Kosmala-Anderson & Wallace, 2013; Row 28; Olsson et al., 2011; Row 30; Ploughman et al., 2012). Being accepted and understood in this way was accompanied by a sense of relief.

Supportive and accepting social relationships were important for pwMS; it felt good to be welcomed by old friends in the same way as they were before MS appeared (In Table 6: Row 28, Olsson et al., 2011; Row 32; Reynolds & Prior, 2003). New friendships could develop too, and MS support groups could be a source of support where “everybody understands and everybody knows” (In Table 6: Row 17, Irvine et al., 2009, p. 4; Row 28; Olsson et al., 2011). Loved ones also needed someone to listen (In Table 6: Row 2, Bogosian et al., 2009; Row 8; DesRosier et al., 1992; Row 18; Jonzon & Goodwin, 2012), and the availability of a good support network (parent, family, and friends) mediated the impact of parental MS for adolescents: ‘You can’t underestimate how much family helps’ (Bogosian et al., 2011, p. 435; Row 3 of Table 6).

### The SDR-Derived Diagram: Getting Out of Negative Cycles

Figure 2 displays how the reciprocal role patterns that emerged out of the analysis of the literature may fit together in ways that affect wellbeing, either positively or negatively. With patterns mapped out in this way, points of exit are more easily identified. Exits afford opportunities for individuals to stop being trapped in dysfunctional cycles of behaviour and relating. At each large arrow in Fig. 2, there is a potential for something to be done differently, i.e., to exit and avoid looping back into unhelpful cycles. All behavioural expressions of the accepting–supported reciprocal role noted above are considered exits from unhelpful patterns, and further to this, we will now discuss specific exit points shown in Fig. 2.

#### a-Exits

The four large arrows with **a-**superscripts highlight the reality that an increase in pwMS’ need for care has the potential to elicit responses from caregivers and family members that are over-protective, intrusive, ignoring, or rejecting in nature and that these relational styles have negative interpersonal and psychosocial consequences for pwMS. Negative reactions from significant others can increase distress whereas supportive reactions can assist adjustment (In Table 6: Row 21, Kralik et al., 2003; Row 27; Olsson et al., 2008; Row 31; Power, 1985), so whenever there are changes in care needs, these must be named and discussed to enable families and pwMS to meet the related challenges and minimise negative outcomes. At times, this process may necessitate support from responsive, accessible health care teams.

#### b-Exits

On the left side of Fig. 2, two large arrows with **b**-superscripts focus on two types of overbearing care, i.e., overprotective care or intrusive care, which can leave the “done to” person feeling controlled by or intruded upon by others. Such experiences are likely to negatively impact pwMS’ wellbeing. Although overbearing care may be well-intentioned, caregiver-pwMS dyads may need help negotiating what support is actually needed and wanted; partners may need to learn to not intervene too soon. Some pwMS may need to learn to “speak up” in tactful ways, to vocalise that even if tasks take twice as long, the sense of accomplishment they experience is helpful to them (Irvine et al., 2009; Row 32 of Table 6). When MS intrudes, pwMS may need support to adapt activities or find alternative meaningful occupations, which can help maintain quality of life and self-esteem (Reynolds & Prior, 2003; Row 32 of Table 6). With respect to helping caregivers behave in ways that are less controlling and less intrusive, family members may need support and encouragement to take time for themselves, to step back and take breaks that allow them to reflect upon and more effectively manage their own behaviours in ways less likely to elicit negative consequences, for themselves and for their loved one with MS.

#### c-Exits

The lower portion of Fig. 2 displays an arrow with a **c**-superscript that focuses directly on pwMS’ behaviours that relate to managing emotional pain and counterproductive reactions that worsen pwMS’ wellbeing. Key strategies when feeling overwhelmed are talking and sharing feelings, yet pwMS often cope by talking to themselves, as reported by almost half of McCabe et al.’s sample (1996; Row 3 of Table 7). Clear communication regarding difficulties and feelings is important (In Table 6: Row 11, Esmail et al., 2007; Row 22; Malcomson et al., 2008) but not easy to do; in fact, women with MS reported their emotional experience feels invisible (Blundell Jones, Walsh, & Isaac, 2014). Psychological interventions could be designed to help pwMS and their families deal more effectively and successfully with relational patterns that otherwise might ensnare pwMS in cycles of interpersonal behaviour that further decrease pwMS’ morale and self-esteem.

#### d-Exits

Arrows with **d**-superscripts in Fig. 2 focus on familial responses to MS that are more negative in nature, namely, ignoring or rejecting interaction patterns, which are very likely to have adverse effects on pwMS’ wellbeing. To reduce the prevalence of these types of reciprocal roles, services could support education for caregivers and family members regarding MS so that understanding and acknowledgement of difficulties is more prevalent in pwMS-caregiver/family relationships. Both pwMS and their families feel that others need to be more informed about MS (Courts et al., 2005, Row 7 of Table 6; Green & Todd, 2008; Row 1 of Table 7), and so programs that increase awareness and understanding at a societal level may be beneficial. Within the family itself, denial, or incomplete acknowledgement of each other’s perspectives, experiences, and emotions leaves pwMS and their family members feeling misunderstood and rejected in their interactions with one another. One strategy to address this problem would be to support pwMS and their families to more successfully share their experience with one another. Esmail et al. (2010; Row 13 of Table 6) noted the importance of a safe, open environment for communication where both partners, one with MS and the other healthy, can address issues as they arise and be able to deal with anything that is brought to the table. Families may require support from providers to develop such open communication particularly if it is something they have struggled with historically.

## Discussion

This integrative review has examined the impact of MS on familial and social relationships and the links between reciprocal role patterns in relationships and wellbeing. Whittemore and Knafl (2005, p. 550) note that the goal of data analysis is to provide “a thorough and unbiased interpretation of primary sources, along with an innovative synthesis of the evidence.” Our application of CAT theory has met this goal; the theory provided a useful way of conceptualising a body of published literature on the effects of MS on social relationships among pwMS, caregivers, and family members that has clinical relevance. CAT highlighted five patterns of relational behaviour within the data set, and the SDR approach enables clinicians to consider practical implications and offer interventions for relationship issues. In this section, we consider service implications; provide a critical analysis of our approach; and consider future directions for research.

MS changes the physical body and influences not only how individuals with MS view and relate to themselves, but also how others view and relate to them. This review identified five reciprocal roles which appear intimately related to the progressive nature of MS, which creates a need for care that increases over time, makes heavy demands on coping ability, and raises issues of dependency like those that occur with other chronic illnesses (e.g., rheumatoid arthritis: Bury, 1982). The unpredictable nature of MS means that pwMS’ care needs can be highly variable. Increases in symptom severity can be temporary, and relapses may be followed by remissions; or symptoms may be enduring with functional losses and progressive deterioration. Every family member is touched by the challenge of dealing with MS (Bowen et al., 2011). The burdens of caregiving can create distance between pwMS and their loved ones (Grytten & Måseide, 2006). Relationships among family members may need “remodelling” (Lyons & Meade, 1995), and if relationship changes are not successfully negotiated, stress increases and mental health can deteriorate for pwMS, and for loved ones.

The reciprocal role relationship patterns we highlighted make a difference for coping, adjustment, and wellbeing; how pwMS and their families respond to the increased need to “be cared for” underpins the link between mental health and chronic illness. Using CAT terminology, Walsh et al. (2000, p. 164) noted that chronic illness can place the affected individual in a child-like relational position of feeling vulnerable and lacking in control. Being “cared for,” can result in pwMS experiencing painful loss of one’s sense of agency, and loss of valued roles. If painful. If others (whether family members, friends or health care providers) behave in ways that are perceived as rejecting or ignoring, or if they “take over,” i.e., are over-protective or intrusive, there is increased emotional pain for the individual with MS.

The literature revealed that pwMS can feel rejected, devalued, and infantilised, and individuals sometimes manage these overwhelming feelings by withdrawing from others, and become isolated. Beal and Stuifbergen (2007, p. 169) noted that “a sense of estrangement from others often accompanies prolonged illness.” This may be related to a pwMS’ sense that others do not understand his or her experience of illness. With reduced social contact, feelings of loneliness and hopelessness increase and the wellbeing of pwMS is negatively affected.

Families are a potent force in pwMS’ adjustment to illness (Power, 1985), and strong social support networks are widely acknowledged to be important for the mental wellbeing of people living with MS (Patrick, Morgan, & Charlton, 1986); it is therefore vital that patterns in interpersonal relationships be considered. This review highlighted that accepting MS-related changes is a key factor in exiting from unhelpful relationship patterns, and an important aspect of effective coping; however, due to the nature of their MS condition, pwMS emphasize that constant re-adjustment and re-acceptance are necessary. It is unclear how much styles of family/couple functioning prior to MS diagnosis influence interpersonal responses to MS. It could be that some families have had an accepting–supported pattern of relating to one another, even prior to illness onset. It is also possible that, at a certain point in the MS journey, for families that have good external support, this reciprocal role pattern may become the more dominant pattern.

### Implications for Service Provision

While research has revealed that MS affects emotional wellbeing (Honarmand & Feinstein, 2009) for a variety of reasons, the role that relationships play cannot be overlooked. Just as the physical and psychological aspects within the individual cannot be separated, and neither can individuals be divorced from the context of relationships in which she or he lives. These factors have major implications for providing services for pwMS. MS services must be more holistic and integrate relatives into care. If MS service systems consider the whole family, clinicians will know how a family is functioning and be able to provide timely family-based interventions when there are difficulties (Fisher & Weihs, 2000; Galushko et al., 2014). Rintell and Melito (2013) suggest that as part of standard care, pwMS and their families should be offered preventive family interventions after diagnosis. Some may question whether services should “interfere” with family life, but pwMS assign very high importance to meeting psychosocial needs such as having good relationships with family (Koopman, Benbow, & Vandervoort, 2006). Moreover, pwMS have indicated that they want support for this area of their lives. Interventions are needed, therefore, that approach families as dynamic units, and that support couples and families to work through unhelpful patterns, to re-find mutuality, and to move towards relating in an accepting–supported way (Badr & Acitelli, 2005; Uccelli, 2014). That said, pwMS should always be consulted about involvement of family members in their care, and conversations around such topics must be handled sensitively.

Health services for persons with chronic illnesses such as MS may need to consider routinely employing psychological practitioners to provide interventions to affected families, couples, and individuals. In the context of ever-reducing budgets this may seem fanciful, but if we consider there is a 45% increase in healthcare costs when co-morbid mental health difficulties are present (Naylor et al., 2012), medical care cannot afford to be divorced from psychosocial issues.

As living with MS is a continual process of adjustment and adaptation, families may need different types of support at different times. Considering the reciprocal roles revealed by our integrative review, we will highlight some examples. Caregivers may benefit from support and find a good balance between giving caring and self-care, which may help them feel that MS is a less negative and intrusive force in their lives, and thereby reduces strain on relationships. Efforts to support pwMS and family members to communicate more effectively with one another and manage feelings will be beneficial. MS service providers must make sure that “emotions are on the agenda,” perhaps using yearly emotional check-ups (Blundell Jones et al., 2014) as part of annual medical reviews. Some families may need assistance to minimise unhelpful overprotection, while pwMS may need support to assert their wishes and opinions regarding independence along with support for finding patterns of successful daily activities that bolster self-esteem. A delicate balance must be struck between independence and dependence, as well as a balance between acknowledging and ignoring of symptoms. Interventions that facilitate such balance and help people find their own solutions will positively influence wellbeing.

### Limitations

By using CAT to interpret the results of this review, attention has been given to ways in which relationships may need remodelling. In this way, the CAT framework has enabled a better connection between the literature review and implications for providing clinical services. Nevertheless, our approach has limitations. A primary consideration is whether the results can be replicated; whilst familiarity with CAT would be required, it is anticipated that similar overarching themes would be revealed. Yet, relationships are complex, and so there may be other pertinent issues or relational difficulties that have not yet been captured by the published literature. Although disease course has been represented in our review (i.e., the number of years living with MS is highly variable), there is a significant lack of longitudinal studies on relationships and coping over time. There is also a possibility that the corpus of published data has been influenced by researcher selectivity and unintended bias.

In this review article, data pertaining to the experiences of multiple individuals was synthesised into a single SDR-derived diagram that covers one illness, and as such speaks to overall themes rather than the intricacies of individual cases. Shannon and Swarbrick (2010) consulted service users to aid the development of a CAT framework for common relational patterns in Bipolar Disorder; similarly, it will be useful to ascertain how those with MS feel about the roles we have highlighted and whether or not those roles automatically apply to every individual. It also will be interesting to consider whether the relationship patterns we have highlighted are similar or different to those for other chronic illnesses. A paper documenting experiences of wives of chronically ill spouses suggests there may be overlap (Eriksson & Svedlund, 2006).

According to Murray (1995), MS care which recognises both neurological and psychosocial issues will be most effective. A condition with so many unpredictable and progressive physical difficulties means psychosocial issues can easily be overlooked, and so MS care must strive to be holistic. Despite the aforementioned limitations, this review has illuminated potential relationship dynamics that may occur in MS and offers a viewpoint from which specific support or interventions can be considered.

## Conclusion

Due to the unpredictability and uncertainty of the MS disease course, along with the strain caused by symptoms, it is understandable that relationships can suffer. Several common patterns of relating were found that can either help or hinder coping and adjustment and affect wellbeing. The relational patterns engaged in by individuals, loved ones, friends, and society in relation to MS are important to understand. Such understanding improves opportunities to revise unhelpful relationship patterns and ameliorate their negative effects. CAT theory has provided a useful framework for deepening understanding of how relationships are affected by chronic illness and has enabled links to be made more readily between a literature review and practice. The presentation of a CAT-informed diagrammatic formulation that represents how patterns interlink has allowed further consideration of patient-focused interventions. Psychological services may have a significant role to play in facilitating understanding and supportive relationships. It will be valuable to ascertain the views of individuals living with MS regarding the reciprocal roles highlighted in this manuscript.
